# Defining the divergent enzymatic properties of RNA polymerases I and II

**DOI:** 10.1074/jbc.RA120.015904

**Published:** 2020-11-24

**Authors:** Ruth Q. Jacobs, Zachariah M. Ingram, Aaron L. Lucius, David A. Schneider

**Affiliations:** 1 Department of Biochemistry and Molecular Genetics, School of Medicine, University of Alabama at Birmingham, Birmingham, Alabama, USA; 2 Department of Chemistry, University of Alabama at Birmingham, Birmingham, Alabama, USA

**Keywords:** gene transcription, RNA polymerase I, RNA polymerase II, enzyme kinetics, RNA synthesis, EC, elongation complex, NLLS, nonlinear least squares, ODEs, ordinary differential equations, rDNA, ribosomal DNA, RNAP, RNA polymerase

## Abstract

Eukaryotes express at least three nuclear DNA-dependent RNA polymerases (Pols) responsible for synthesizing all RNA required by the cell. Despite sharing structural homology, they have functionally diverged to suit their distinct cellular roles. Although the Pols have been studied extensively, direct comparison of their enzymatic properties is difficult because studies are often conducted under disparate experimental conditions and techniques. Here, we directly compare and reveal functional differences between *Saccharomyces cerevisiae* Pols I and II using a series of quantitative *in vitro* transcription assays. We find that Pol I single-nucleotide and multinucleotide addition rate constants are faster than those of Pol II. Pol I elongation complexes are less stable than Pol II elongation complexes, and Pol I is more error prone than Pol II. Collectively, these data show that the enzymatic properties of the Pols have diverged over the course of evolution, optimizing these enzymes for their unique cellular responsibilities.

In contrast to prokaryotic cells, which express a single RNA polymerase, eukaryotes express at least three nuclear RNA polymerases (Pols I, II, and III) (1, 2, 3). Since the discovery of three distinct Pols and their fundamental properties (1, 4, 5), the transcriptional roles of the Pols have been further elucidated (6). Pol I synthesizes rRNA, Pol II synthesizes mRNA and most regulatory RNA, and Pol III synthesizes the 5 S rRNA and tRNA. Although the eukaryotic Pols are structurally similar (7), these enzymes have evolved distinct roles within the cell, and we suggest they have also acquired biochemical properties to suit their specialized roles (8).

Pol I is localized to the nucleolus where it transcribes ribosomal DNA (rDNA). The rDNA in *Saccharomyces cerevisiae* is organized into a single genetic locus consisting of approximately 150 tandem 9.1 kb repeats on chromosome XII. Pol I synthesizes a single transcript from each repeat, producing the 35 S pre-RNA that is cotranscriptionally and posttranscriptionally modified to form the 25 S, 18 S, and 5.8 S rRNAs. Together with the 5 S rRNA (synthesized by Pol III), these RNAs form the backbone of the eukaryotic ribosome (9, 10). Despite having only one target gene, far fewer than those for Pols II and III, Pol I activity accounts for approximately 60% of transcription in a growing yeast cell (11, 12).

Pol II is localized in the nucleus and is responsible for transcribing approximately 6000 protein-coding genes, far more loci than for either Pol I or Pol III (13). The average length of Pol II-derived transcripts is approximately 3 kb (14, 15). Pol II transcription is also influenced by the largest number of transcription factors (16, 17); approximately 60 polypeptides are recruited to promote efficient transcription elongation, RNA processing, RNA export, and chromatin remodeling (16, 18). As Pol II transcribes the largest subset of genes and diversity of promoters (19), the variety of recruited protein factors is advantageous, as it allows for gene-specific transcriptional control and regulation (20).

Pol III is localized in the nucleus and is responsible for a diverse set of noncoding RNAs including tRNAs, 5 S rRNA, U6 snRNA, and several microRNAs and snoRNAs (21). The average length of Pol III-synthesized products is approximately 100 bases long (22). Interestingly, while Pol III is responsible for the shortest transcripts of the Pols, Pol III is the largest of the three nuclear Pols with 17 subunits compared with 14 and 12 for Pols I and II, respectfully (8, 20, 23). Similarly to Pol I, Pol III activity is tightly coordinated with cell growth and proliferation as it synthesizes the 5 S rRNA required to form a eukaryotic ribosome (24).

The differences between the Pols in subunit composition, localization, transcript demand, regulatory mechanisms, and RNA products illustrate the different selective pressures that have been exerted on these enzymes during eukaryotic evolution (25, 26).

By expressing three unique nuclear RNA polymerases, eukaryotic cells gain the capacity for more complex control of gene expression. For example, unique transcription factors can regulate the expression of individual genes by modulating the activity and environmental response of a specific polymerase without influencing the other two polymerases (27). We hypothesize an additional advantage to having multiple Pols. The enzymatic properties of each polymerase have evolved to optimally transcribe its unique target genes.

To test this idea, we utilized quantitative *in vitro* transcription assays to compare several intrinsic enzymatic properties of Pols I and II using a fully purified system. Pols I and II exhibit divergent properties in every assay performed. Nucleotide addition catalyzed by Pol II was found to be slower compared with Pol I. However, Pol II displays higher transcription elongation complex (EC) stability and much lower misincorporation activity. Together, all of these findings support the hypothesis that unique selective pressures have driven the divergence of DNA-dependent RNA polymerases, resulting in enzymes whose transcription elongation properties are suited to their cellular roles.

## Results

### Single-nucleotide addition by Pol I is faster than by Pol II

**Figure 1:**
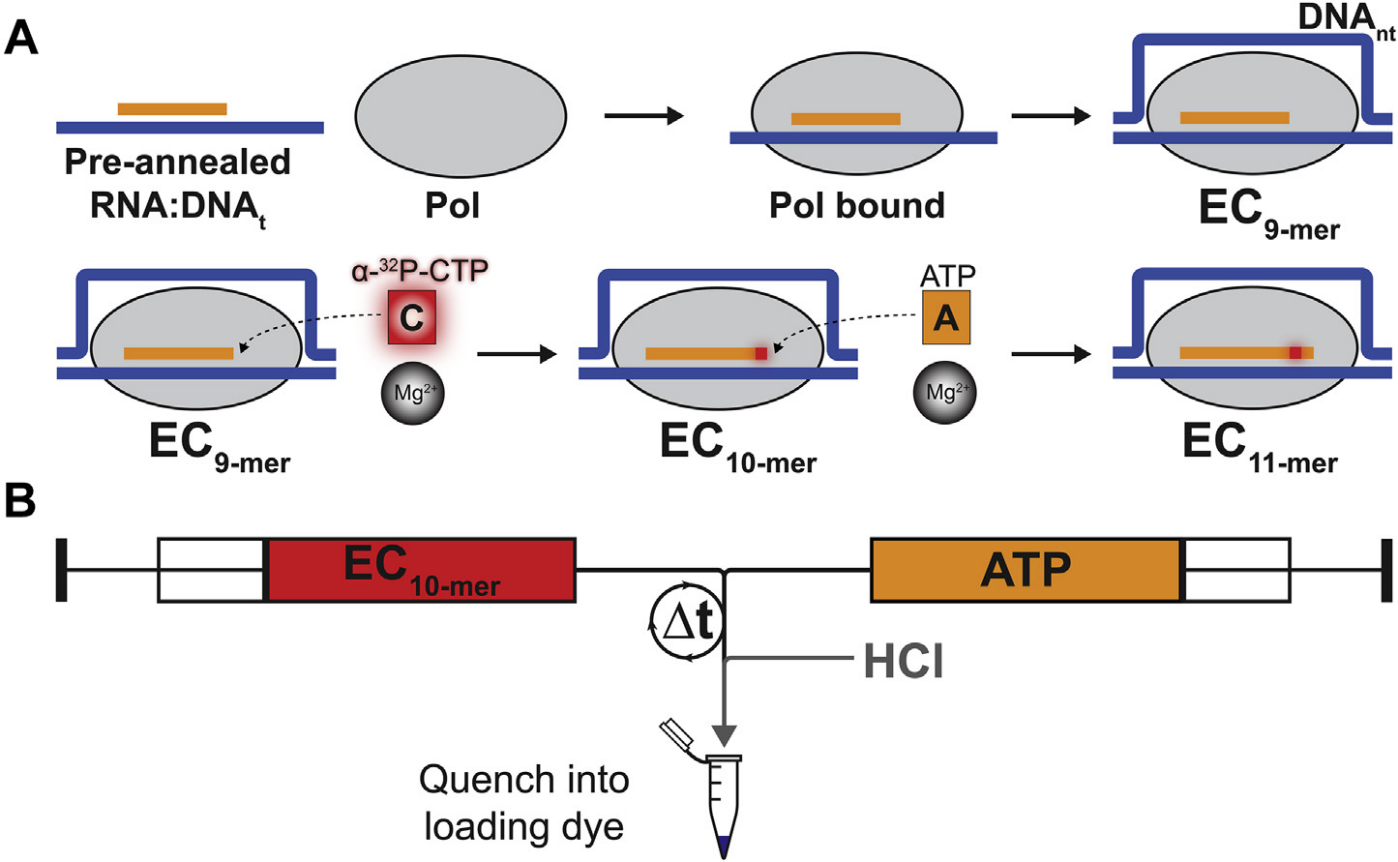
**Promoter-independent *in vitro* transcription assay schematic and chemical quench-flow setup.***A*, experimental process to assemble and radioactively label elongation complexes (ECs) and subsequently observe a single-nucleotide incorporation event. *B,* diagram of the chemical quench-flow. Radiolabeled ECs are rapidly mixed 1:1 with the NTP substrate. Time points are collected between 0.005 and 10 s.

To quantitatively compare the constants that govern nucleotide addition catalyzed by both Pol I and Pol II, we employed a promoter-independent *in vitro* transcription assay, previously developed to describe Pol I (28). The EC was formed in buffer A by first binding Pol I or II to a preannealed RNA:DNA_t_ hybrid, then adding the nontemplate DNA strand (Fig. 1*A*). ECs were radiolabeled by incubation with α-^32^P-CTP and the cofactor Mg^2+^ (Fig. 1*A*). ECs with a radiolabeled 10-mer RNA were rapidly mixed with the next cognate nucleotide, ATP, and stopped at fixed reaction times (0.005–10 s) using a chemical quench-flow (Fig. 1*B*).

Time courses of Pols I and II single-nucleotide addition were collected in triplicate at a saturating concentration of ATP (1 mM). Samples were run on polyacrylamide gels, exposed to phosphorimager screens, and analyzed.

**Figure 2:**
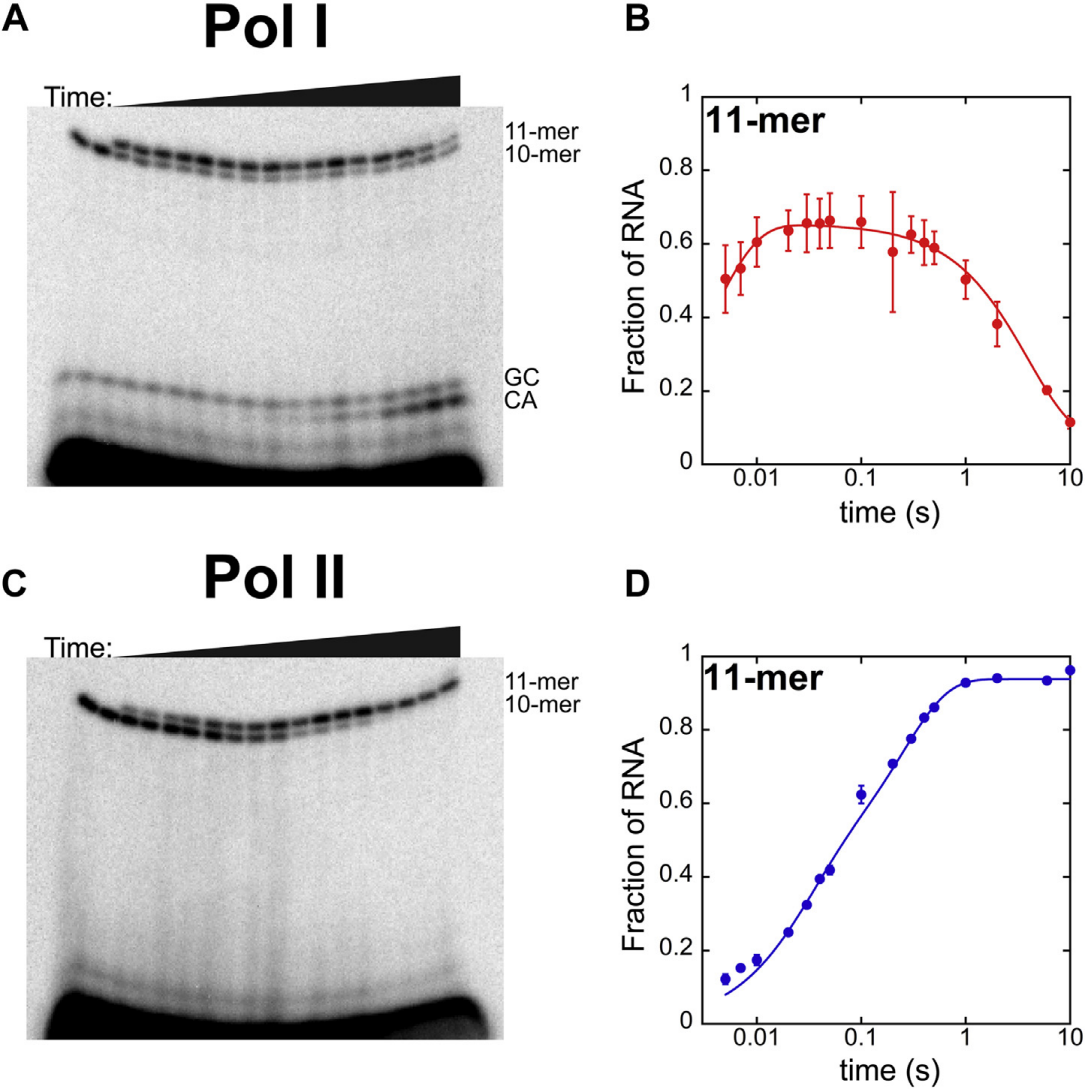
**Single-nucleotide addition time courses for Pols I and II.***A,* 28% denaturing urea PAGE gel resolving RNA species from single-nucleotide addition experiment at 1 mM ATP by Pol I. *B,* Pol I 11-mer time course. Fraction of 11-mer RNA as a function of time quantified using Equation 1. *C,* Pol II single-nucleotide addition PAGE gel at 1 mM ATP. *D,* Pol II 11-mer time course. Fraction of 11-mer RNA as a function of time quantified according to Equation 2. Each point represents the mean of three independent reactions with error bars corresponding to the standard deviation about the mean. Pol, nuclear RNA polymerase.

For Pol I, we observed bands on the gels consistent with a 10-mer (the α-^32^P-CTP nascent RNA generated during the labeling step), an 11-mer (the 10-mer extended by AMP addition), and because of Pol I’s intrinsic nuclease activity, a GC and a CA dimer (Fig. 2*A*). The GC dimer was produced from the 10-mer RNA during labeling, and the CA dimer resulted from cleavage of 11-mer RNA during the nucleotide incorporation reaction (Fig. 2*A*).

(1) $$Fraction\;of\;RNA=A_{fast}\left(\exp\left(-k_{obs,fast}\times t\right)\right)+\;A_{cleavage}\left(exp\left(-k_{obs,cleavage}\times t\right)\right)$$

Pol I single-nucleotide addition time courses displayed a rise in the fraction of 11-mer RNA and subsequent decrease because of RNA cleavage (Fig. 2*B*). These data were fit to a sum of two exponentials to describe single-nucleotide addition, which is governed by rate constants k_obs,fast_ and k_obs,cleavage_ (Equation 1, Fig. 2*B*). This strategy, whereby single-nucleotide addition data are fit to a sum of two exponentials, has been successfully used previously to describe Pol I’s nucleotide incorporation mechanism (28).

Conversely, for Pol II, we observed only two RNA species, a 10-mer and 11-mer RNA, but no cleavage products (Fig. 2*C*). Pol II lacks intrinsic nuclease activity; thus, this was expected.

(2) $$Fraction\;of\;RNA=A_{fast}\left(1-\exp\left(-k_{obs,fast}\times t\right)\right)+\;A_{slow}\left(1-exp\left(-k_{obs,slow}\times t\right)\right)\;$$

Pol II single-nucleotide addition time courses displayed a rise and subsequent plateau of the fraction of 11-mer RNA (Fig. 2*D*). Weighted fits were performed using both one exponential (Equation S1) and the sum of two exponentials (Equation 2). When fit to a single exponential equation, we observed a systematic deviation between the best fit line and the experimental data (Fig. S1). Therefore, it was necessary to fit to the sum of two exponentials to appropriately describe the experimental data.

The observed rate constant governing single-nucleotide addition by Pol I, k_obs,fast_, was (200 ± 100) s^-1^, whereas the RNA cleavage rate constant, k_obs,cleavage_, was much slower at (0.19 ± 0.01) s^-1^. Pol II single-nucleotide addition was described by two rate constants, determined to be (38 ± 9) s^-1^ and (4 ± 0.6) s^-1^ for k_obs,fast_ and k_obs,slow_, respectively. Pols I and II exhibit dramatically different nucleotide addition kinetics under these conditions. Furthermore, only one rate constant is required to describe nucleotide addition by Pol I (29), whereas Pol II nucleotide addition is governed by at least two unique rate constants. This finding is consistent with previous work published by Bustamante *et al.* that proposed two kinetic steps, NTP sequestration and bond formation, follow NTP binding (30). Ongoing studies are focused on a quantitative analysis of these steps to define the kinetic mechanism of Pol II nucleotide addition.

### Multinucleotide addition by Pol I is faster than by Pol II and displayed more heterogeneous rate constants

Pol I exhibited faster nucleotide addition kinetics than Pol II for a single AMP incorporation. To test whether Pol I was faster than Pol II over the course of multiple nucleotide incorporation events, we added saturating concentrations of both ATP and GTP to our reactions, resulting in nine successive incorporation events. Thus, in addition to quantifying the rate of multinucleotide incorporation, this approach allowed us to evaluate the effect of DNA sequence on nucleotide addition kinetics.

**Figure 3:**
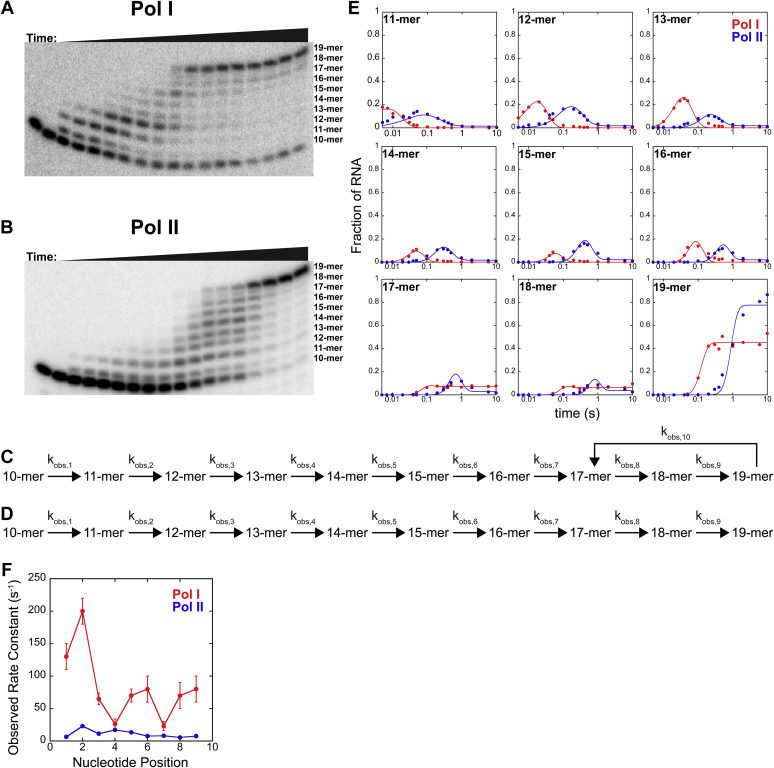
**Pol I and II multinucleotide addition time courses.** 28% denaturing urea PAGE gels resolving RNA species from multinucleotide addition experiment by Pol I (*A*) and Pol II (*B*) at 1 mM ATP and 1 mM GTP. *C,* Scheme 1 describes Pol I multinucleotide addition. *D,* Scheme 2 describes Pol II multinucleotide addition. (*E*) Pols I and II representative data sets of each RNA species over time fit to their respective schemes. *F,* Plot of Pols I and II individual k_obs_ values for each parameter k_obs,1_ − k_obs,9_. Average k_obs_ values were (53 ± 4) s^−1^ for Pol I and (8.6 ± 0.1) s^−1^ for Pol II. Each point represents the mean of three independent reactions with error bars corresponding to the standard deviation about the mean. Pol, nuclear RNA polymerase.

Time courses for Pols I and II were collected using the chemical quench-flow in the presence of 1 mM ATP and 1 mM GTP in buffer A (Fig. 3, *A–B*). From these time courses, we measured the appearance and disappearance of each RNA intermediate over time. To analyze and compare individual incorporation events by Pols I and II, a minimal kinetic model was developed using Scheme 1 and Scheme 2 for Pols I and II, respectively (Fig. 3, *C–D*). The additional kinetic parameter, k_obs,10_, was included to describe the intrinsic cleavage activity of Pol I conferred by its A12.2 subunit (31, 32). This step is not required for Pol II because the transcription elongation factor TFIIS is required for nascent RNA cleavage (33), and TFIIS was absent from these reactions. Parameter values were optimized using a MATLAB toolbox called MENOTR (Methods), and the corresponding best fit lines are shown with the experimental data in Fig. 3*E*. Representative data sets composed of nine different time courses describing the abundance of each RNA intermediate for Pols I and II were compared (Fig. 3*E*). Experimental data sets consisted of nine time courses describing the abundance of each RNA intermediate (11-mer through 19-mer) were fit simultaneously. The mean and standard deviation between replicates were calculated for each of the parameters of Schemes 1 and 2 (Table 1). Plots of k_obs_ values for Pols I and II at each nucleotide addition step showed that Pol II was systematically slower at all nine nucleotide incorporation positions (Fig. 3*F*). The average k_obs_ for Pol I was measured to be (53 ± 4) s^-1^, whereas the average k_obs_ for Pol II was (8.6 ± 0.1) s^-1^. These values were consistent with the trend observed in Fig. 2, as Pol II incorporated nucleotides slower than Pol I.

**Table 1:** Resultant parameter values from Pol I and II multinucleotide addition time courses fit to Schemes 1 and 2, respectively

Parameter	Pol I	Pol II
	Best fit value	Best fit value
k_1_ (s^-1^)	130 ± 20	6.4 ± 0.6
k_2_ (s^-1^)	200 ± 20	23 ± 2
k_3_ (s^-1^)	65 ± 9	11.3 ± 0.6
k_4_ (s^-1^)	26 ± 8	17.1 ± 0.6
k_5_ (s^-1^)	70 ± 10	13.5 ± 0.6
k_6_ (s^-1^)	80 ± 20	7.7 ± 0.3
k_7_ (s^-1^)	23 ± 7	8.2 ± 0.3
k_8_ (s^-1^)	70 ± 20	5.5 ± 0.4
k_9_ (s^-1^)	80 ± 20	7.6 ± 0.4
k_10_ (s^-1^)	12 ± 3	NA
Fraction of active Pols	0.52 ± 0.06	0.79 ± 0.02

Pol, nuclear RNA polymerase.

Pol I multinucleotide addition time courses were fit globally to Scheme 1 and the resultant mean and standard deviation of the optimized parameters are shown. Similarly, Pol II multinucleotide addition time courses were fit to Scheme 2, and the mean and standard deviation on each parameter is shown.

The rate constants of Pol I multinucleotide were more variable, with k_obs_ values that ranged from (23–200) s^-1^, compared with (5.5–17.1) s^-1^ for Pol II (Table 1). Thus, nucleotide addition by Pol I is influenced by the identity of the NTP encoded and/or the template DNA sequence context more than Pol II. This finding suggests that the template DNA impacts the catalytic properties of Pols I and II differently.

### Pol II elongation complexes are more stable than those of Pol I

Our data revealed dramatic differences in nucleotide addition kinetics for Pols I and II. We questioned whether other enzymatic properties of the ECs also diverged. EC stability is a critical property of a polymerase; ECs for the individual Pols must be sufficiently stable to processively transcribe each gene, but the complexes must be unstable enough to efficiently terminate transcription or release the DNA at lesions.

**Figure 4:**
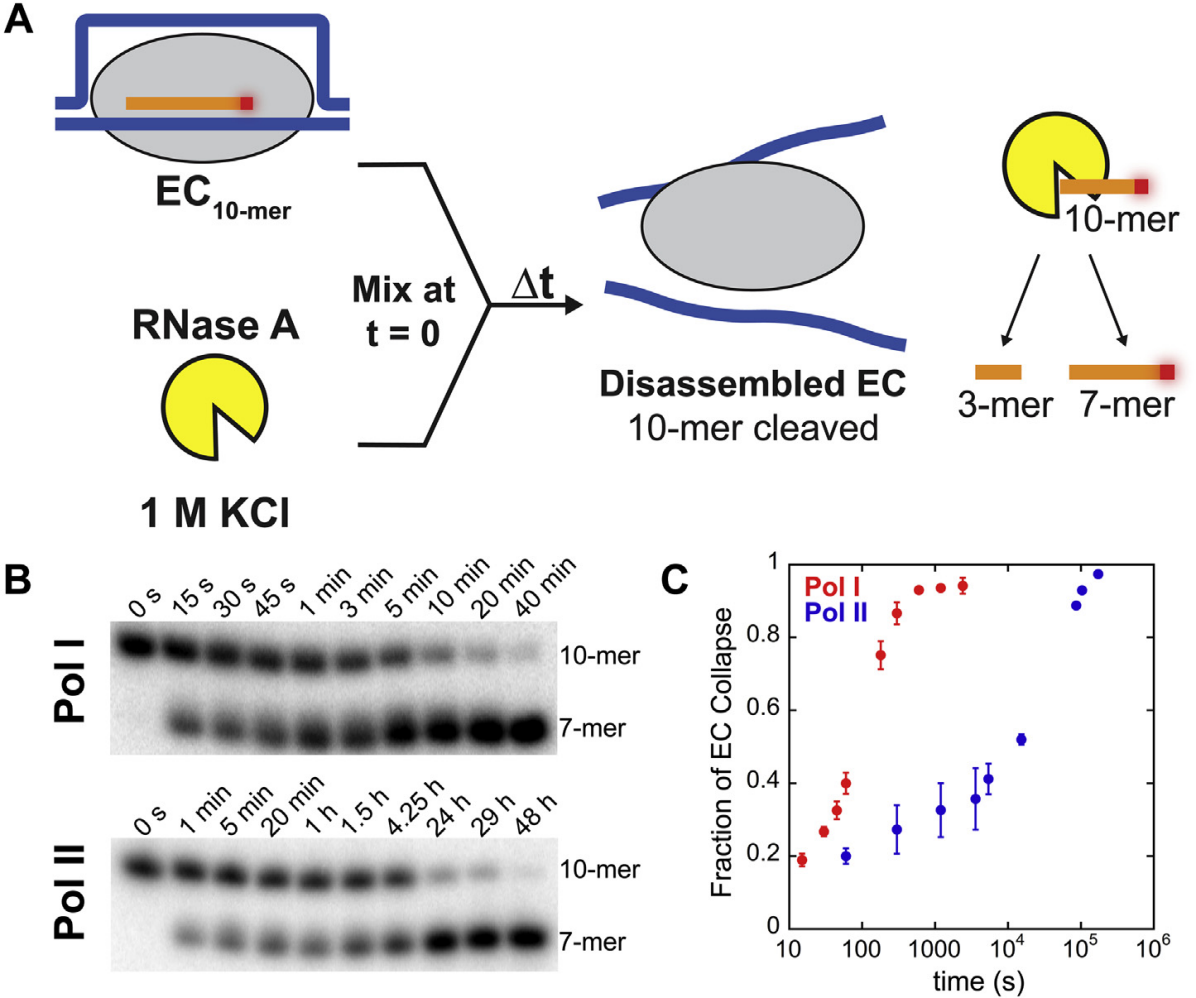
**EC stability assay schematic and experimental results for Pols I and II.***A,* EC stability experimental schematic. Radiolabeled ECs are mixed 1:1 with 1 M KCl and 10 μM RNase A at t = 0. Reactions are collected continuously and quenched in loading dye. *B,* 10-mer, protected RNA, and 7-mer, unprotected cleaved RNA, RNA species are resolved on a denaturing polyacrylamide gel over Pol I and II time courses. *C,* quantification of disassembled ECs over time for Pols I and II. Each point represents the mean of three independent reactions with error bars corresponding to the standard deviation about the mean. EC, elongation complex; Pol, nuclear RNA polymerase.

We previously developed an RNase protection assay for Pol I that quantifies the stability of stalled ECs on a DNA:RNA hybrid (34). ECs were formed in buffer A and radiolabeled by the same method used in our promoter-independent *in vitro* transcription assay (Fig. 1*A*). Radiolabeled ECs were mixed with RNase A in a destabilizing salt concentration (1 M KCl), and reactions were carried out with the reaction time spanning from 0 s to 40 min for Pol I and 0 s to 48 h for Pol II. (Fig. 4*A*). As intact ECs protected the 10-mer RNA from cleavage, accumulation of cleaved RNA indicated EC collapse over time.

We found that almost all Pol I ECs collapsed within 40 min, whereas Pol II required 48 h to release the DNA (Fig. 4*B*). The observation that Pol II EC collapse was dramatically right-shifted compared with Pol I indicates that Pol II forms a much more stable EC (Fig. 4*C*). The difference between the EC stabilities of Pols I and II suggests that they have diverged over time to suit their individual roles. It is known that Pol II transcribes nucleosome-bound templates, whereas the rDNA template for Pol I is thought to be nucleosome-free or loosely bound by histones. Perhaps this difference in template provided a selective pressure toward a more stable EC for Pol II.

### Pol I is more error-prone than Pol II

**Figure 5:**
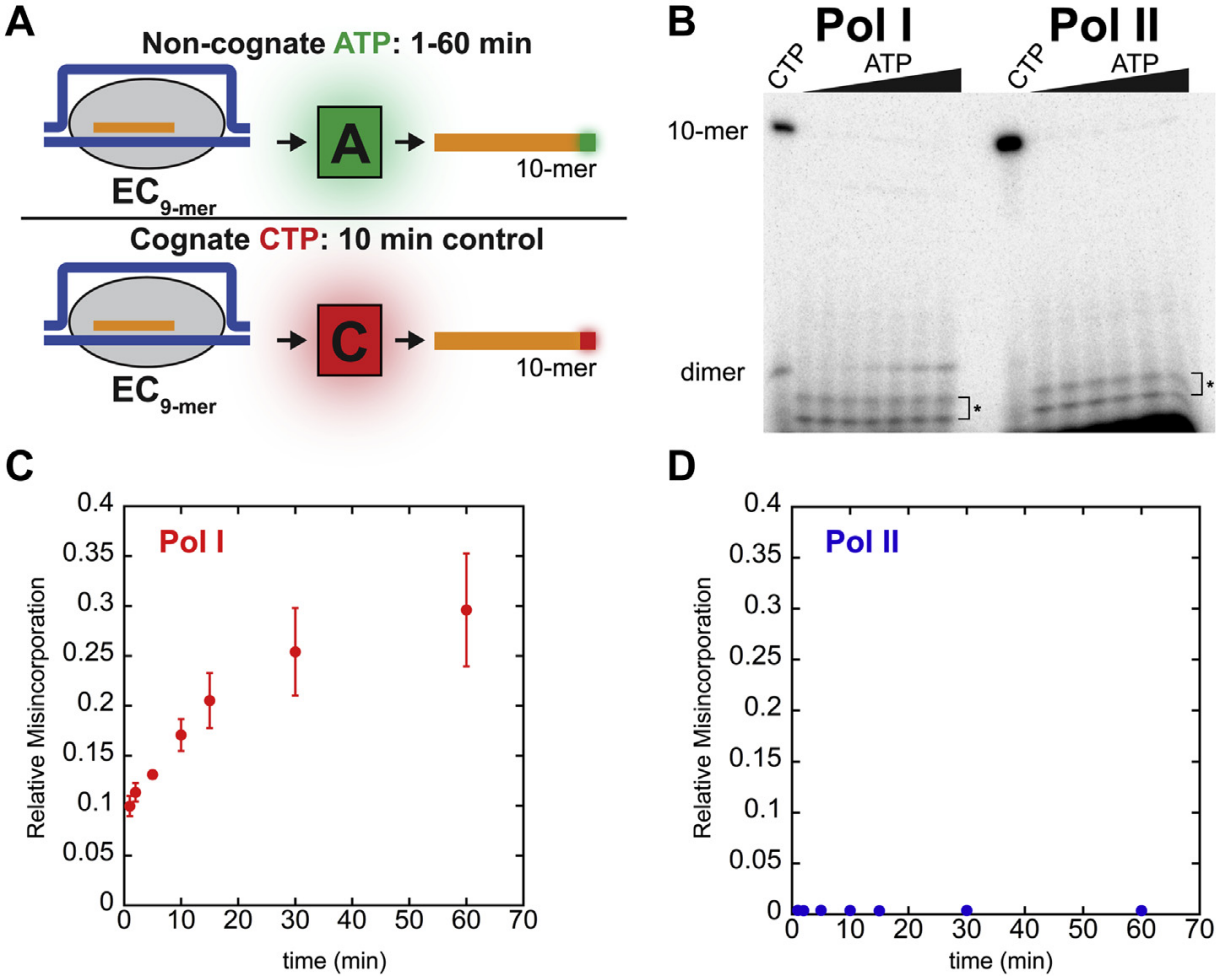
**Misincorporation assay schematic and experimental results for Pols I and II.***A,* misincorporation assay experimental schematic. Pol I or II ECs are incubated with α-^32^P-CTP or α-^32^P-ATP to detect correct or incorrect incorporation, respectively. *B,* representative gel of Pol I and II CTP control and misincorporation time courses. Dimer species produced by Pol I is indicated. Correct incorporation of α-^32^P-CTP (representing active ECs) is compared with misincorporation of α-^32^P-ATP to quantify number of misincorporation events per active EC for Pol I. ∗ denotes impurities present in α-^32^P-ATP (*C*) and Pol II (*D*). Each point represents the mean of three independent reactions with error bars corresponding to the standard deviation about the mean. EC, elongation complex; Pol, nuclear RNA polymerase.

Nucleotide addition by Pol I was significantly faster than by Pol II. It has been suggested that faster incorporation may lead to reduced fidelity (35, 36). Thus, we sought to compare misincorporation by Pol I to Pol II. To test this hypothesis, we employed a previously described misincorporation assay (29), using identical synthetic DNA:RNA hybrid templates. ECs were formed in buffer A and incubated with either the next cognate nucleotide, α-^32^P-CTP, or a noncognate nucleotide, α-^32^P-ATP (Fig. 5*A*). The RNAs generated from either correct incorporation or misincorporation are compared with measure relative misincorporation by Pols I and II.

For the CTP control, Pol I incorporated α-^32^P-CTP to yield a 10-mer RNA and a small population of those 10-mers were cleaved and resolved as dimers (Fig. 5*B*). Quantification of total 10-mer and dimer species intensity resulted in a value reflective of number of active ECs in the reaction. Upon incubation of the noncognate α-^32^P-ATP, Pol I misincorporated, extending to a 10-mer, and subsequently cleaved two nucleotides from the 3’ end of the RNA, resulting in a dimer species (Fig. 5*B*). The intensity of 10-mer and dimer species resulting from misincorporation was summed, and the summation was divided by the intensity of the abundance of RNA produced by correct incorporation of CTP. This procedure yields a relative misincorporation value of the number of misincorporation events per active EC.

Over a 60 min incubation with α-^32^P-ATP Pol I ECs produced up to 0.30 ± 0.06 misincorporation events per active EC (Fig. 5*C*). Misincorporation was clearly evident in the accumulation of the dimer species, but the rate of misincorporation remains low (∼0.005 events per EC per minute). It is important to note that this value is also not reflective of misincorporation events by Pol I in the presence of all NTPs. Rather, this value is indicative of the maximal relative misincorporation per Pol I EC over a long incubation when only a noncognate nucleotide is provided.

For Pol II, correct and incorrect incorporation are only indicated by the presence of a 10-mer RNA species, because Pol II lacks intrinsic cleavage activity (Fig. 5*B*). Pol II incorporated the correct nucleotide, α-^32^P-CTP, but misincorporation during incubation with α-^32^P-ATP was almost undetectable (Fig. 5*B* and *D*). Relative misincorporation was quantified as described for Pol I but only summing the amount of 10-mer for correct and incorrect incorporation events. Pol II produced only 0.003 ± 0.002 misincorporation events per active EC during 60 min incubation with noncognate NTP (Fig. 5*D*). These data demonstrate that the fidelity of nucleotide addition is substantially different for Pols I and II.

### Buffer conditions influence nucleotide addition by Pols I and II

**Figure 6:**
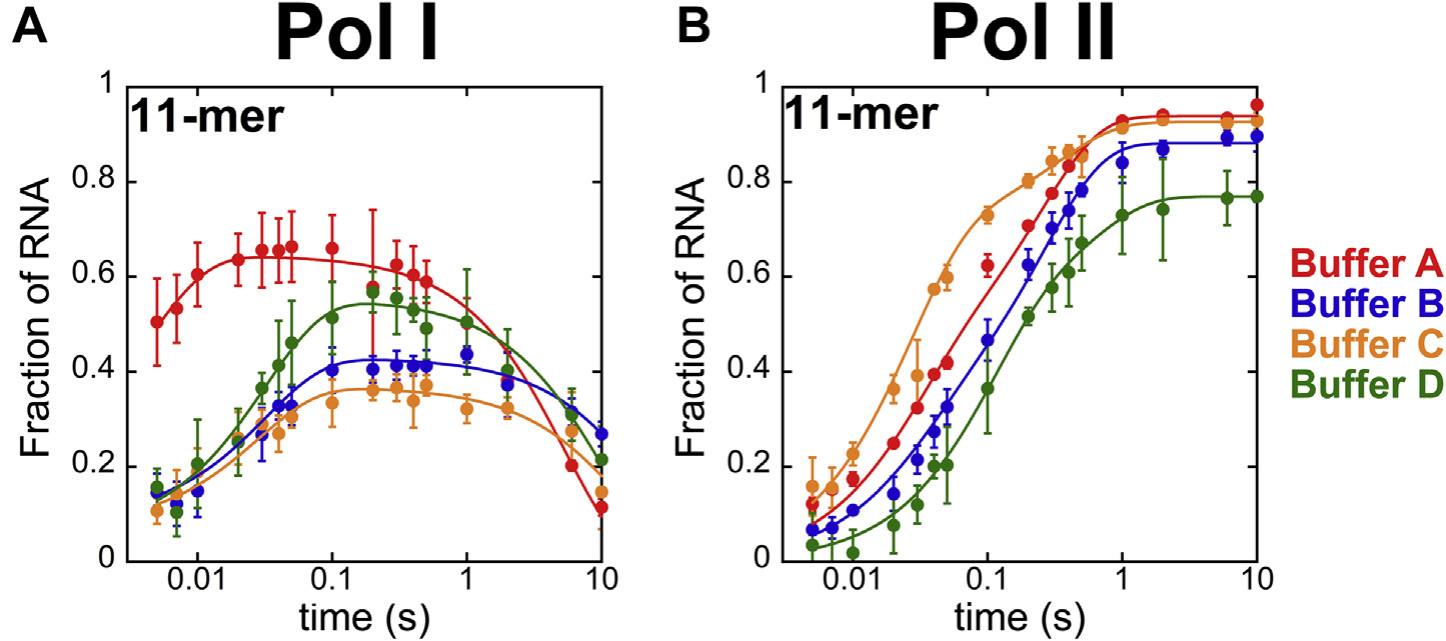
**Single-nucleotide addition time courses catalyzed by Pols I and II in buffers A–D.** Plots of Pol I (*A*) and Pol II (*B*) 11-mer RNA appearance over time. Best fit lines describing Pol I and Pol II data sets are shown as lines based on Equations 1 and 2, respectively. Each point represents the mean of three independent reactions with error bars corresponding to the standard deviation about the mean. Pol, nuclear RNA polymerase.

It is possible that these studies revealed overall faster nucleotide incorporation rate constants for Pol I based on a reaction condition, buffer A, that was optimized for Pol I (Fig. 2). To test whether buffer choice influenced the comparison of the enzymes, we repeated single-nucleotide addition measurements in four different reaction buffers (A–D, Methods). Buffer A was used for Figure 2, Figure 3, Figure 4, Figure 5. Buffers B and C were used in previous studies focused on Pol II characterization, and buffer D is a pH-adjusted version of buffer A. The time courses in buffers A–D were collected in triplicate, and the mean and standard deviation of the fraction of 11-mer species at each data point were recorded (Fig. 6*A*). The Pol I and II time courses were fit identically as for Fig. 2 using Equations 1 and 2, respectively.

The fastest rate constant governing nucleotide addition by Pol I was observed in buffer A, ∼200 s^-1^, whereas the slowest was observed in both buffer B and buffer D, ∼30 s^-1^ (Table 2). However, buffer B, C, and D all yielded nucleotide incorporation rates that were within error of one another. The Pol I data collected in buffer A also had the fastest cleavage rate constant of ∼0.19 s^-1^ (Table 2). The time courses collected in buffers B and C both exhibited slower cleavage rate constants. Buffer D is identical to buffer A, except it is pH adjusted to match buffer B. In buffer D, the cleavage rate constant for Pol I was faster than in buffers B and C, whereas nucleotide incorporation rate remained similar to that observed in buffers B and C. Thus, Pol I nucleotide addition was more pH-dependent than nuclease activity.

**Table 2:** Resultant parameter values from Pol I time courses fit to a sum of two exponentials

Pol I				
Parameters	Buffer A	Buffer B	Buffer C	Buffer D
k_obs,fast_ (s^-1^)	200 ± 100	31 ± 4	35 ± 7	31 ± 4
k_obs,cleavage_ (s^-1^)	0.19 ± 0.01	0.047 ± 0.007	0.07 ± 0.01	0.10 ± 0.01
A_fast_	−0.05 ± 0.76	−0.34 ± 0.02	−0.29 ± 0.02	−0.5 ± 0.03
A_cleavage_	0.646 ± 0.009	0.430 ± 0.009	0.37 ± 0.01	0.55 ± 0.01

The exponential equation, Equation 1, was optimized to describe single-nucleotide addition by Pol I**.** Optimized parameters were determined using MENTOR with parameter error generated from 500 Monte-Carlo simulations.

Pol II single-nucleotide incorporation was also analyzed in buffers A–D. The data were collected in triplicate, and the mean was calculated for each data point. The Pol II time courses displayed the mean values of the fraction of 11-mer RNA as a function of time and were fit with Equation 2 (Fig. 6*B*). Contrary to what we observed with Pol I, where nucleotide addition is described only by k_obs,fast_, Pol II nucleotide addition is governed by both k_obs,fast_ and k_obs,slow_. Pol II single-nucleotide rate constants, k_obs,fast_ and k_obs,slow_, were within error across buffers A, B, and C (Table 3). Nucleotide addition was slowest in buffer D, with rate constants calculated to be ∼10 s^-1^ and ∼1.8 s^-1^ for k_obs,fast_ and k_obs,slow_, respectively.

**Table 3:** Resultant parameter values from Pol II time courses fit to a sum of two exponentials

Pol II				
Parameters	Buffer A	Buffer B	Buffer C	Buffer D
k_obs,fast_ (s^-1^)	38 ± 9	35 ± 9	38 ± 6	10 ± 21
k_obs,slow_ (s^-1^)	4 ± 0.6	3.8 ± 0.4	3 ± 2	1.8 ± 0.8
A_fast_	0.4 ± 0.06	0.28 ± 0.05	0.7 ± 0.06	0.5 ± 0.1
A_slow_	0.54 ± 0.06	0.6 ± 0.04	0.23 ± 0.06	0.27 ± 0.1

The exponential equation, Equation 2, was optimized to describe single-nucleotide addition by Pol II**.** Optimized parameters were determined using MENTOR with parameter error generated from 500 Monte-Carlo simulations.

To compare nucleotide addition rate constants between Pols I and II over the four buffers tested, Pol I k_obs,fast_ and Pol II k_obs,slow_ values were evaluated. With all chemical reactions, the observed rate constant is determined by the slowest kinetic step. Accordingly, the nucleotide addition cycle is rate-limited by the slowest step in a single-nucleotide addition cycle. Pol I’s RNA cleavage rate constant, k_obs,cleavage_, is a separate enzymatic activity that is preceded by nucleotide addition and, therefore, does not limit the nucleotide addition cycle. Thus, the rate-limiting step of Pol I nucleotide addition is k_obs,fast_. Pol II’s slowest step in single-nucleotide addition is k_obs,slow_.

The slowest step of single-nucleotide addition by Pol I was consistently and substantially faster than Pol II’s slowest step across buffers A–D, ranging from ∼50 to ∼8 fold faster than k_obs,slow_ values of Pol II, depending on the buffer used (Tables 2 and 3). Pol I and II single-nucleotide addition rate constants were substantially lower in buffer D compared with the other three buffers tested. Buffer A and buffer D are identical except for pH. To test whether pH influenced multinucleotide addition catalyzed by Pols I and II, we performed reactions with saturating ATP and GTP in buffer D (Fig. S2*A*). The lower pH in buffer D reduced the rate constants for nucleotide addition by both enzymes at every position in the template, and the amplitude of the reduction was approximately the same at all positions (Fig. S2*B*). These data support our previous conclusion that Pol I is faster than Pol II and demonstrate that nucleotide addition by both enzymes is sensitive to pH.

## Discussion

### Evolutionary differences between Pols I and II

This study examined several key enzymatic properties of both Pols I and II, revealing substantial divergence between these two enzymes in every assay performed. We suggest that these properties arose under selective pressure that favored the optimization transcription elongation. In other words, the biochemical characteristics of each polymerase have diverged to meet the unique demands of their separate roles within the cell. Pol I is faster at single-nucleotide and multinucleotide addition, more prone to EC collapse, has a higher misincorporation activity and is more sensitive to changes in reaction conditions than Pol II. Showing that in the absence of Pol-specific transacting factors, each polymerase displayed fundamentally unique enzymatic properties.

### Absence of A12.2 subunit renders Pol I “Pol II-like”

Intriguing similarities emerged when we compared our Pol II nucleotide addition results to previous characterization of a Pol I isoform lacking the A12.2 subunit (ΔA12.2) (32). As mentioned above, the A12.2 subunit confers Pol I’s intrinsic cleavage activity, whereas Pol II requires a dissociable factor, TFIIS. We previously characterized two consequences of A12.2 absence. As expected, ΔA12.2 failed to cleave RNA, and, interestingly, there was a significant alteration in the mechanism of ΔA12.2 nucleotide incorporation. Compared with wild-type Pol I single-nucleotide addition, which is described by k_obs,fast_ alone (Fig. 2, Equation 1) (28), ΔA12.2 required a fast and slow rate constant, k_obs,fast_ and k_obs,slow_, respectively, to describe the data (32). Notably, the mechanism of ΔA12.2 nucleotide addition and observed rate constants are similar to our data describing Pol II single-nucleotide addition (Fig. 2, Equation 2).

This comparison provides insight into the evolution of three structurally and functionally distinct nuclear eukaryotic Pols from the ancestral RNA polymerase (RNAP) shared by Bacteria, Archaea, and Eukarya (37). Bacterial RNAP is substantially divergent functionally and structurally from the archaeal and eukaryotic RNAPs. Bacterial RNAP requires σ-factors for initiation, whereas archaeal and eukaryotic RNAPs use TATA-binding protein and transcription factor IIB (37). Eukaryotic Pols and archaeal RNAP share a heterodimer stalk structure that is absent in bacterial RNAP (38). Therefore, eukaryotic Pols are more similar to archaeal RNAP than bacterial RNAP (39). Pol II is the most similar to archaeal RNAP, as they have similar subunit composition and utilize homologous transcription factors. This suggests Pol II emerged as the first eukaryotic polymerase (39). A key similarity between Pol II (33), archaeal RNAP (40), and bacterial RNAP (41) is that they all require a transacting transcription factor to elicit RNA cleavage activity. The integration of polypeptides or transcription factors as permanent subunits drove the specialization of early eukaryotic Pols (37). Specifically, the addition of *bona fide* cleavage factors by Pols I and III, A12.2 and C11, respectively, led to structurally distinct Pols with a substantial functional impact on misincorporation, backtrack recovery, elongation rate, and reinitiation (42, 43, 44, 45).

### Functional importance of rapid Pol I elongation rate constants

Pol I was found to incorporate single and multiple nucleotides substantially faster than Pol II (Figs. 2 and 3). If this is observed *in vivo,* then each engaged Pol I would yield more rRNA over time. This outcome would appear to be beneficial, because during periods of rapid growth, there is robust demand for new ribosome synthesis. However, there would be extensive functional consequences of this enhanced rRNA synthesis rate. Because rRNA synthesis is tightly coupled to rRNA processing (46), the complex rRNA processing machinery must be equally efficient to match the rate of rRNA synthesis by Pol I. The mechanisms by which transcription elongation by Pol I and pre-rRNA processing are coupled remain largely elusive. Furthermore, how cells co-regulate Pols I, II, and III for efficient expression of ribosomes remains a topic of interest (47).

### Pols I and II differ in misincorporation amplitude, not transcriptional error rate

Our data showed that misincorporation by Pol I was roughly two orders of magnitude greater than for Pol II under these reaction conditions (Fig. 5). It is important to note that the observed relative misincorporation is not synonymous with final transcriptional error frequency. Interestingly, the final transcriptional error rates for Pols I and II were shown to be similar, with an error rate of 4.3 x 10^-6^ per base for Pol I and 3.9 x 10^-6^ per base for Pol II (48). How can these rates be justified with the value reported here? As previously published (34, 49, 50) and noted above, Pol I carries a subunit (A12.2) that confers robust RNA cleavage activity. On the other hand, Pol II lacks intrinsic cleavage activity, relying on activation of cleavage by TFIIS. We suggest that A12.2 enhances proofreading by Pol I (by cleavage of nascent RNA and recovery from stalled ECs because of misincorporation). As a consequence, the fidelity of rRNA synthesis is similar to that of mRNA synthesis, despite faster and more error prone nucleotide addition by Pol I. If correct, this assertion would also rationalize the incorporation of A12.2 as a *bona fide* subunit for Pol I *versus* reliance on transacting factors.

### Importance of EC stability and instability for Pols I and II *in vivo*

Differences in the templates for Pols I and II *in vivo* may have presented different selective pressures on Pol EC stability. We observed that ECs formed by Pol I are much less stable than those assembled by Pol II (Fig. 4). Pol I is densely packed on the rDNA, and this high density of ECs increases the risk for EC pileups or "traffic jams" in the event of a pause or lesion (51, 52). It is reasonable to think that these EC pileups would result in impaired RNA synthesis (53) and potentially DNA damage, because it is known that replisome and RNAP collisions induce DNA breaks (54). To avoid this, polymerases must be able to disengage from the DNA efficiently to minimize pileups that could lead to replisome–Pol or Pol–Pol collisions. Thus, the density of Pol I per gene may have selected for the decreased EC stability observed here.

Compared with the nucleosome-free DNA template for Pol I (55, 56, 57), Pol II transcribes DNA templates that are protected by regularly spaced nucleosomes (58, 59) and must remain stably bound during nucleosomal displacement/remodeling to transcribe the entirety of the template (60). It is well-characterized that Pol II undergoes promoter-proximal pausing after it initiates and encounters the first nucleosome (61, 62) and that its release is critically linked to efficient elongation (63). Therefore, transient pausing by Pol II is a critical point for transcriptional regulation (50, 63, 64). It is reasonable to conclude that both of these properties of mRNA coding genes require that Pol II form a more stable EC.

### Difference between our data and previous reports

There is great variability in the reported Pol elongation rates in the literature, especially for Pol II (30, 45, 65, 66, 67, 68, 69, 70, 71). Our results showed how different reaction conditions are likely responsible for the diversity of resultant parameters (Fig. 6). However, one consistent trend exists: Pol II is slower than Pol I (30, 45), which is corroborated in this study. A previous study by Grill *et al.* that evaluated transcription elongation by both Pols I and II reported faster transcription elongation by Pol I than Pol II, (39.2 ± 2.5) nt/s *versus* (24.6 ± 2.6) nt/s (45). Here, we report a much greater difference between Pols I and II, (53 ± 4) nt/s *versus* (8.6 ± 0.1) nt/s (Fig. 3). The variation of elongation rates is likely because of the fact that they observed elongation over very long templates, > 800 bp, which increases the frequency of pausing and misincorporation by Pol I, ultimately decreasing the apparent net elongation rate. Additionally, we utilized a variety of reaction conditions to reveal their impact on nucleotide addition kinetics (Fig. 6).

### Unique properties of Pol I present vulnerabilities for selective inhibition

Pol I has emerged as a validated anticancer target (72, 73, 74). Ribosome biogenesis is upregulated in highly proliferative cancer cells. It is thought that transcription of the rDNA is rate-limiting for ribosome biogenesis; thus, it is a key target for therapeutic inhibition. A decrease in the synthesis of rRNA by Pol I would result in fewer ribosomes, thereby interfering with the rapid cell growth and proliferation inherent to most cancer cells. Therapeutic inhibition of Pol I must be specific and not inhibit the other polymerases. To inhibit Pol I without affecting other closely related enzymes, it is critical to reveal vulnerabilities unique to Pol I. Discovery of these vulnerabilities of Pol I will contribute to studies aimed at optimizing Pol I-specific anticancer therapeutics.

BMH-21 is an example of a compound identified for its repressive effects on rRNA synthesis and derivatives of BMH-21 are in preclinical development as a cancer therapeutic (73). BMH-21 intercalates into GC-rich and nucleosome-free DNA, a central feature of rDNA, thereby disrupting Pol I activity. It is possible that its localization to the rDNA is not the sole explanation for the specificity of BMH-21. Perhaps BMH-21 is found throughout the genome, not solely the rDNA, but does not affect Pols II or III in the same manner. Our data revealed that ECs formed by Pol II are far more stable than those of Pol I (Fig. 4). This finding suggests that Pol II may be more capable of traversing through obstacles, such as BMH-21 intercalated into the template, whereas Pol I is more sensitive and undergoes transcriptional arrest. This model offers a plausible explanation to why BMH-21 specifically affects Pol I. Throughout this study, we characterized fundamental properties of the nuclear Pols, revealing novel enzymatic differences that will provide a foundation for designing and understanding clinically valuable Pol I-specific inhibitors.

## Experimental procedures

### Polymerase purification

#### Yeast strains

The yeast strain for Pol I purification was described previously (75). For Pol II purification, a yeast strain was constructed with the second largest subunit, RPB2, C-terminally tagged with a TEV cleavage site, three HA repeats, and 10 histidine residues.

#### Protein purification

Yeast strains expressing tagged Pols I and II were grown at the Bioexpression and Fermentation Facility, Department of Biochemistry and Molecular Biology at University of Georgia. The Pols were purified from approximately 150 g of wet cell mass. The cells were washed and lysed using a cell disrupter in breakage buffer (400 mM (NH_4_)_2_SO_4_, 50 mM Tris-SO_4_ pH 7.8, 10 mM MgCl_2_, 10 μM ZnCl_2_, 10% glycerol). Supernatant was loaded on five 5 ml Ni columns (GE Healthcare) following ultracentrifugation (Ti45 rotor, 32,000 rpm, 4 °C, 1 h). Ni columns were washed in KCl low imidazole buffer (100 mM KCl, 50 mM Tris-SO4 pH 7.6, 5 mM MgCl2, 10 mM imidazole, 20% glycerol) and subsequently eluted directly onto a heparin column (GE Healthcare) attached downstream with KCl high imidazole buffer (same as KCl low imidazole but with 250 mM imidazole). Ni columns were removed, and heparin column was washed into low salt KCl buffer without imidazole (100 mM KCl, 20 mM HEPES pH 7.8, 1 mM MgCl_2_, 10 μM ZnCl_2_, 20% glycerol) and eluted with high salt KCl buffer (same as low KCl buffer but 2 M KCl) into a 50 ml conical tube. Eluate was run over desalting columns (GE Healthcare) to change to MonoQ suitable buffers (A: 200 mM KOAc, 20 mM HEPES pH 7.8, 1 mM MgCl_2_, 10 μM ZnCl_2_, 10% glycerol; B: same as A but 2 M KOAc). Flow-through was collected and manually loaded onto a MonoQ column (GE Healthcare). Fractionation was performed using a 0 to 100% buffer B gradient at a flow rate of 0.25 ml/min over 100 min. Pol fractions identity and purity were confirmed with Coomassie-stained SDS PAGE gels and Western blots.

### *In vitro* transcription assays

#### Buffers

The four buffers under investigation include:

**Table:** 

A (28)	B (71)	C (45)	D (buffer A pH adjusted to match buffer B)
20 mM Tris-OAc pH 7.9	50 mM HEPES pH 7.5	20 mM HEPES pH 7.6	20 mM Tris-OAc pH 7.5
40 mM KCl	80 mM KOAc	60 mM (NH_4_)_2_SO_4_	40 mM KCl
10 mM MgOAc	5 mM MgSO_4_	8 mM MgSO_4_	10 mM MgOAc
2 mM DTT	10 mM DTT	10 μM ZnCl_2_	2 mM DTT
0.2 mg/ml BSA	10% glycerol	10% glycerol	0.2 mg/ml BSA

#### Single-nucleotide addition *in vitro* transcription assay

Single-nucleotide addition assays utilized to investigate Pols I and II were first described in the supplemental of a previously published manuscript (28). Buffer A is used in Fig. 2 and compared with three additional buffers, B–D, in Fig. 6. ECs are formed by incubating Pol I or II with a preannealed RNA:DNA_t_ and the DNA_nt_ strand. The EC is labeled with the addition of α-^32^P-CTP and Mg^2+^. The labeling reaction is stopped with an excess nonradioactive CTP and EDTA. Labeled ECs are loaded into a 1 ml syringe and loaded opposite of the substrate syringe, consisting of the next cognate nucleotide, ATP, and Mg^2+^, in the chemical quench-flow. The chemical quench-flow rapidly mixes the ECs and substrate mix in a 1:1 ratio for a programmed amount of time and stops the reaction with the addition of 1 M HCl. Varying reaction times (0.005–10 s) are achieved by different length loops of the quench-flow. Additional HCl and neutralization buffer is added to each quenched reaction to yield equal volumes of each time point and to neutralize the HCl. An aliquot of the reaction mixture is transferred to the RNA loading dye and subsequently boiled and loaded on a 28% polyacrylamide denaturing urea gel with 1X TBE running buffer.

### EC stability assay

EC stability assays for Pols I and II were executed essentially as described previously (34). ECs are formed in buffer A and labeled as described for the single-nucleotide addition assay. At t = 0, labeled ECs are mixed in equal amount with 10 μM RNase A (Worthington Biochemical, Lakewood, NJ) and 1 M KCl. Aliquots of reaction mixture are removed for each time point and quenched in RNA loading dye. Samples are boiled, loaded, and ran on a 28% polyacrylamide denaturing urea gel with 1X TBE running buffer in the top (anode) buffer reservoir and 1X TBE with 1 M NaOAc in the bottom (cathode) buffer reservoir to resolve dimer species.

### Misincorporation assay

Misincorporation assay for Pol I and II was described previously (29). ECs for Pols I and II are formed with the incubation of the RNA:DNA_t_ and DNA_nt_ strand in buffer A. The EC mix is split, one to measure correct incorporation, the other to measure misincorporation. The EC mix aliquots was either incubated with α-^32^P-CTP and Mg^2+^, to observe correct incorporation, or α-^32^P-ATP and Mg^2+^, to observe misincorporation. Time points were collected and quenched in RNA loading dye. Samples were boiled and ran on a 28% polyacrylamide denaturing urea gel with 1X TBE for the top (anode) buffer reservoir and 1X TBE with 1 M NaOAc in the bottom (cathode) buffer reservoir to resolve dimer species. To control for variable radioactive intensity of the α-^32^P-CTP and α-^32^P-ATP stocks, a series of dilutions were spotted on Whatman paper, and a ratio of intensity was calculated.

#### Multinucleotide addition *in vitro* transcription assay

Multinucleotide addition assays were set up identical to the single-nucleotide addition assays previously described (28), with the addition of 1 mM GTP to the substrate mix in buffer A in Fig. 3 and in buffer D in Fig. S2.

### Model-dependent quantifications

#### Parameter optimization

MENOTR, Multi-start Evolutionary Nonlinear OpTimizeR, is a custom-built hybrid nonlinear least squares (NLLS) genetic algorithm which is useful in optimizing parameters describing a wide variety of biochemical problems. Traditional NLLS methods exhibit an initial guess dependence which can result in optimization routines stalling in a local solution. The genetic algorithm component of MENOTR addresses this issue by starting a variety of starting points and subsequently performing NLLS on partially refined initial guesses. MENOTR also has the capabilities to perform Monte Carlo and grid search error analysis. An original manuscript is currently being written to outline MENOTR’s capabilities. MENTOR was used throughout this manuscript to optimize parameters describing Pols I and Pol II transcription kinetics.

KaleidaGraph (Synergy Software, Reading, PA) was also used in this manuscript to perform parameter optimization. KaleidaGraph has the benefit of being a quick and easy method to perform simple parameter optimization problems.

#### Model-dependent analysis

To optimize a set of parameters in a given scheme, MENOTR requires the user to input the set of ordinary differential equations (ODEs) describing each species in the scheme. The set describing Scheme 1 are 1.1-1.10, and the set describing Scheme 2 are 2.1-2.10. MENOTR used the sets of ODEs in the built in MATLAB function ODE23tb to numerically simulate a set of time courses for each species. The time courses are then separated based on their respective RNA species component and summed. The reason for this is because some intermediates are not able to be separated in this experimental strategy. MENOTR then optimizes the parameters and reports the set of parameters that describe the data best.

(1.1) $$\frac{d\left[10mer\right]}{dt}=-\left[10mer\right]k_{1}$$

(1.2) $$\frac{d\left[11mer\right]}{dt}=\left[10mer\right]k_{1}-\left[11mer\right]k_{2}$$

(1.3) $$\frac{d\left[12mer\right]}{dt}=\left[11mer\right]k_{2}-\left[12mer\right]k_{3}$$

(1.4) $$\frac{d\left[13mer\right]}{dt}=\left[12mer\right]k_{3}-\left[13mer\right]k_{4}$$

(1.5) $$\frac{d\left[14mer\right]}{dt}=\left[13mer\right]k_{4}-\left[14mer\right]k_{5}$$

(1.6) $$\frac{d\left[15mer\right]}{dt}=\left[14mer\right]k_{5}-\left[15mer\right]k_{6}$$

(1.7) $$\frac{d\left[16mer\right]}{dt}=\left[15mer\right]k_{6}-\left[16mer\right]k_{7}$$

(1.8) $$\frac{d\left[17mer\right]}{dt}=\left[16mer\right]k_{7}-\left[17mer\right]k_{8}+\left[19mer\right]k_{10}$$

(1.9) $$\frac{d\left[18mer\right]}{dt}=\left[17mer\right]k_{8}-\left[18mer\right]k_{9}$$

(1.10) $$\frac{d\left[19mer\right]}{dt}=\left[18mer\right]k_{9}-\left[19mer\right]k_{10}$$

(2.1) $$\frac{d\left[10mer\right]}{dt}=-\left[10mer\right]k_{1}$$

(2.2) $$\frac{d\left[11mer\right]}{dt}=\left[10mer\right]k_{1}-\left[11mer\right]k_{2}$$

(2.3) $$\frac{d\left[12mer\right]}{dt}=\left[11mer\right]k_{2}-\left[12mer\right]k_{3}$$

(2.4) $$\frac{d\left[13mer\right]}{dt}=\left[12mer\right]k_{3}-\left[13mer\right]k_{4}$$

(2.5) $$\frac{d\left[14mer\right]}{dt}=\left[13mer\right]k_{4}-\left[14mer\right]k_{5}$$

(2.6) $$\frac{d\left[15mer\right]}{dt}=\left[14mer\right]k_{5}-\left[15mer\right]k_{6}$$

(2.7) $$\frac{d\left[16mer\right]}{dt}=\left[15mer\right]k_{6}-\left[16mer\right]k_{7}$$

(2.8) $$\frac{d\left[17mer\right]}{dt}=\left[16mer\right]k_{7}-\left[17mer\right]k_{8}$$

(2.9) $$\frac{d\left[18mer\right]}{dt}=\left[17mer\right]k_{8}-\left[18mer\right]k_{9}$$

(2.10) $$\frac{d\left[19mer\right]}{dt}=\left[18mer\right]k_{9}$$

## Data availability

All data described are contained within the manuscript.

## Conflict of interest

The authors declare that they have no conflicts of interest with the contents of this article.
