# Supplementary material for: Defining the divergent enzymatic properties of RNA polymerases I and II
Source: J Biol Chem. 2020 Nov 24;296:100051. doi: 10.1074/jbc.RA120.015904 (PMC7948988; doi:10.1074/jbc.RA120.015904)
Supplement: Supplementary data [file mmc1.pdf]

## SUPPLEMENTARY DATA

$Fraction\ of\ RNA = A\left(1 - exp(-k_{obs} \times t)\right)$ Supplementary Eq. 1

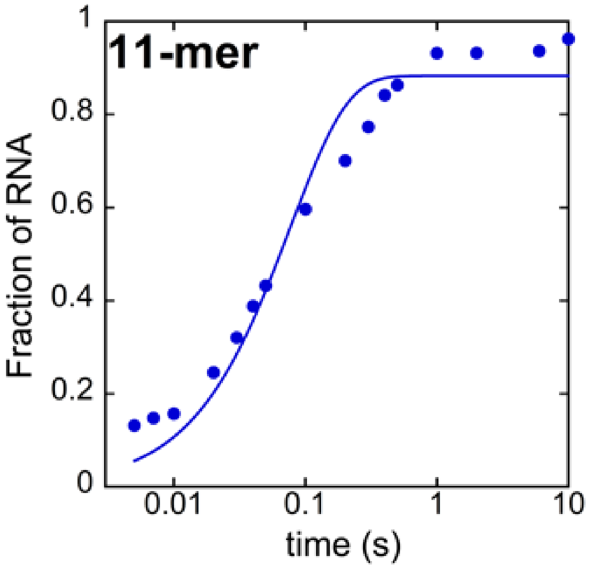


**Supplementary Figure 1. Pol II single-nucleotide addition time course fit to a single exponential equation.** A) Pol II single-nucleotide addition time course collected at 1 mM ATP fit to Supplementary Eq. 1 fails to describe data.

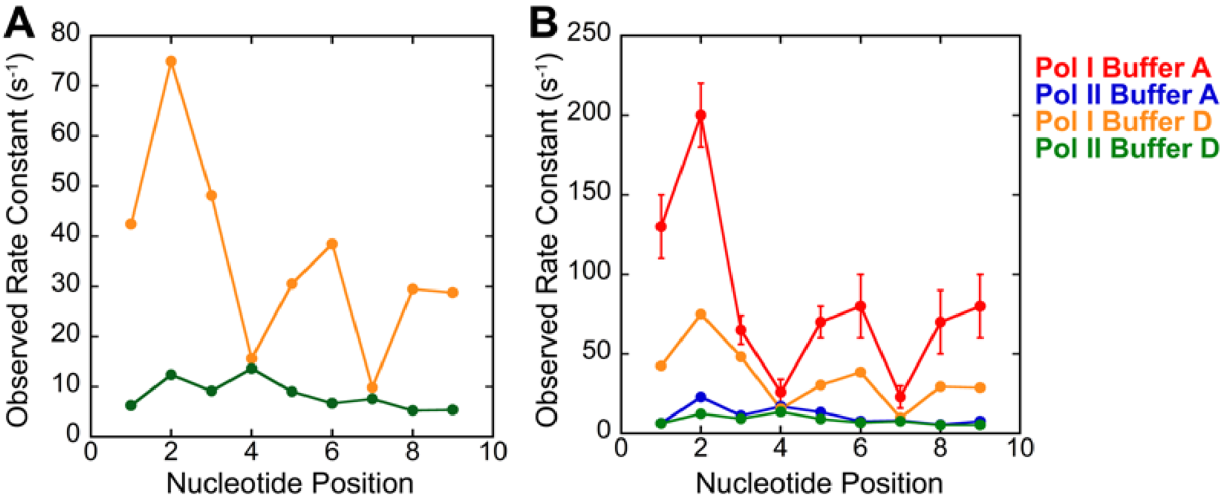


**Supplementary Figure 2. Pol I and II multi-nucleotide addition in buffer D.** A) Plot of Pols I and II individual $k_{obs}$ values for each parameter $k_{obs,1}$ - $k_{obs,9}$ in buffer D. B) Plot of Pols I and II individual $k_{obs}$ values for each parameter $k_{obs,1}$ - $k_{obs,9}$ in buffer A (Figure 3F) and D.
